# ‘Monkey yodels’—frequency jumps in New World monkey vocalizations greatly surpass human vocal register transitions

**DOI:** 10.1098/rstb.2024.0005

**Published:** 2025-04-03

**Authors:** Christian T. Herbst, Isao T. Tokuda, Takeshi Nishimura, Sten Ternström, Vicky Ossio, Marcelo Levy, W. Tecumseh Fitch, Jacob C. Dunn

**Affiliations:** ^1^ Department of Behavioral and Cognitive Biology, University of Vienna, Vienna, Austria; ^2^ Center for the Evolutionary Origins of Human Behavior, Kyoto University, Inuyama, Japan; ^3^ Department of Mechanical Engineering, Ritsumeikan University, Kusatsu, Japan; ^4^ Graduate School of Human Sciences, The University of Osaka, Suita, Japan; ^5^ Department of Speech, Music and Hearing, School of Electrical Engineering and Computer Science, KTH Royal Institute of Technology, Stockholm, Sweden; ^6^ La Senda Verde, North Yungas, Bolivia; ^7^ Behavioural Ecology Research Group, Anglia Ruskin University, Cambridge CB1 1PT, UK; ^8^ ENES Bioacoustics Research Laboratory, University of Saint-Etienne, St-Etienne 42023, France

**Keywords:** vocal membrane, laryngeal mechanism, call repertoire, NLP vocalization, fundamental frequency contol

## Abstract

We investigated the causal basis of abrupt frequency jumps in a unique database of New World monkey vocalizations. We used a combination of acoustic and electroglottographic recordings *in vivo*, excised larynx investigations of vocal fold dynamics, and computational modelling. We particularly attended to the contribution of the vocal membranes: thin upward extensions of the vocal folds found in most primates but absent in humans. In three of the six investigated species, we observed two distinct modes of vocal fold vibration. The first, involving vocal fold vibration alone, produced low-frequency oscillations, and is analogous to that underlying human phonation. The second, incorporating the vocal membranes, resulted in much higher-frequency oscillation. Abrupt fundamental frequency shifts were observed in all three datasets. While these data are reminiscent of the rapid transitions in frequency observed in certain human singing styles (e.g. yodelling), the frequency jumps are considerably larger in the nonhuman primates studied. Our data suggest that peripheral modifications of vocal anatomy provide an important source of variability and complexity in the vocal repertoires of nonhuman primates. We further propose that the call repertoire is crucially related to a species’ ability to vocalize with different laryngeal mechanisms, analogous to human vocal registers.

This article is part of the theme issue ‘Nonlinear phenomena in vertebrate vocalizations: mechanisms and communicative functions’.

## 1. Background

Vocalizations are generated by a system of coupled oscillators [1–3] with inherent non-linearities, capable of producing vibratory phenomena such as subharmonics or deterministic chaos ([4–6]; see also [7]). These irregularities are typically called nonlinear phenomena (NLP). One specific class of NLP is abrupt transitions between distinct oscillatory states of the laryngeal voice source. Such NLP are brought about by bifurcations, i.e. abrupt and quantal change of a dynamic system from one oscillatory state to another, brought about by only a small change of boundary conditions or parameter values [5,8,9]. Among other effects, bifurcations can result in abrupt variations in the oscillatory or fundamental frequency ( $f_{o}$ ). These abrupt $f_{o}$ transitions, termed ‘ $f_{o}$ jumps’ in this text, can be found, for example, in human yodelling and in the ‘voice breaks’ of adolescent males. They are also present in various kinds of non-human animal vocalizations—see [10,11] for further details.

Notably, $f_{o}$ jumps are found in the vocalizations of a number of non-human primates (see the electronic supplementary material, table S1 for selected sources), sometimes even with repeated alternations between the low- $f_{o}$ and high- $f_{o}$ state, analogous to human yodelling. However, these interesting vocal phenomena have not, to date, received much systematic attention in bioacoustic research. In particular, little is known about the principles underlying the mechanisms of their production.

**Figure 1.**
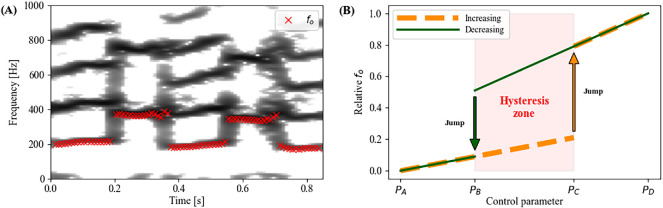
(A) Spectrogram visualization of human ‘yodelling’ phonation with repeated abrupt $f_{o}$ jumps. (B) Schematic illustration of a hysteresis phenomenon during vocalization, where $f_{o}$ jumps occur at different control parameter values depending on previous conditions (increasing versus decreasing)—inspired by [16]. See [10] for a deeper discussion of the hysteresis phenomenon.

$f_{o}$ jumps are a well-documented feature of the human voice [12–15] (see figure 1A for an example). There, the distinct oscillatory states of the laryngeal system are often identified as ‘vocal registers’ (see [17] for an in-depth review), a concept originating in historic voice pedagogy [18]. While registers may be discussed on purely acoustic or even perceptual terms, the key driving factors are different oscillatory states of the vocal folds, constituted by distinct laryngeal vibratory mechanisms [19]. Even though the exact number of vocal registers in humans is still debated [17,20], a consensus seems to have emerged about two main registers: (i) laryngeal mechanism M1, also called ‘chest register or ‘modal’ register; and (ii) laryngeal mechanism M2, also called ‘falsetto register’ or sometimes ‘head voice’. While M2 is predominantly used in singing (and only rarely in speech), M1 is also used as the main mechanism for speech vocalizations—cf. [21]. The main difference between these two laryngeal mechanisms is that M1 is predominantly produced at a lower $f_{o}$ with a strong rotational vibratory mode of the vocal folds, while M2 is typically produced at a higher $f_{o}$ without a pronounced rotational mode (see [10] for details). Typical $f_{o}$ ratios when abruptly changing between M1 and M2 were found to be in the range of 1.2 to 2.5 [16].

Given the largely comparable vocal anatomy between humans and non-human primates and other mammals, it is surprising that vocal registers have not received more attention in animal bioacoustics. Apart from a recent discovery of vocal registers in odontocetes (toothed whales), produced in their nasal vocal organ [22], only sparse information is available for non-human mammals (see [10] for further details). Based on non-invasive electroglottographic (EGG) evidence, Herbst *et al*. showed that the Japanese macaque’s ‘coo’ call is analogous to the ‘falsetto’ or M2 laryngeal mechanism in humans. Also using EGG evidence, Brown & Cannito hypothesized that the Syke’s monkey’s squeal is analogous to the ‘modal’ register [23] or laryngeal mechanism M1 in humans. Finally, investigating acoustic signals, Riede & Zuberbühler suggested that the Diana monkey alarm call is produced with a ‘pulse-register [24], potentially analogous to ‘vocal fry’ (or laryngeal mechanism M0) in humans [25].

Notably, non-human primates possess an additional oscillating laryngeal structure which has been lost during human evolution, i.e. a thin, low-mass vocal membrane situated cranially along the top margin of the vocal fold. This structure has recently been identified as a key component in the laryngeal voice generation of non-human primates [26]. Based on previous theoretical considerations [26,27], we hypothesize here that—given its low mass—this additional oscillator might be capable of better facilitating $f_{o}$ transitions, enabling distinct laryngeal vibratory mechanisms, analogous to human voice registers. Here, we investigate $f_{o}$ bifurcations in the voice production mechanism of non-human primates using New World monkeys as models. We employ a multi-disciplinary approach involving anatomy, *in vivo* and *ex vivo* evidence, and mathematical modelling to document the function of vocal membranes for producing register-like abrupt $f_{o}$ transitions in non-human primate vocalizations. Of all the non-human primates studied, the New World monkeys have evolved the tallest vocal membranes [26], suggesting an important role for them in their vocal production.

## 2. Methods

### (a) Data acquisition

We investigated vocal production in New World monkeys using a range of methods described below. Twelve individuals representing six species were examined originally *in vivo*. Of these, three species systematically produced abrupt $f_{o}$ jumps. We also documented vocal anatomy and *ex vivo* vocal production mechanisms in a subsample of these species.

#### (i) Anatomy

Micro Computed tomography (CT) scans from two genera, *Ateles paniscus* (female, Pr2446) and *Sapajus apella* (male, Pr2746), were from [26]. The larynges were preserved in 10% formalin at the Japan Monkey Centre. The prepared specimens were scanned at the Center for the Evolutionary Origins of Human Behaviour, Kyoto University [26], using diffusible iodine-based contrast-enhanced CT (diceCT) [28].

#### (ii) *In vivo* data acquisition

Data were acquired at the La Senda Verde animal refuge, a non-profit organization located in the tropical region east of the Bolivian Andes. Non-invasive recordings were made *in vivo* of spontaneous vocalizations from 12 hand-reared New World monkeys, representing six different species (*Alouatta caraya, Alouatta sara, Ateles chamek, Cebus albifrons, Saimiri boliviensis* and *Sap. apella*). An overview of all investigated animals is given in the electronic supplementary material, table S2. The *in vivo* research was carried out with full approval from Senda Verde Animal Refuge. All animal housing and handling at Senda Verde are in accordance with Bolivian law. During the study, we adhered to the ‘Guidelines for the treatment of animals in behavioral research and teaching’ [29]. Research methods were approved by ARU ethics committee in accordance with GSP guidelines (ARU: ETH2324−2894).

Data were acquired in a temporary laboratory with an area of approximately 100 m ${,}^{2}$ . Because this room had no sound-proofing, typical ambient forest noise was audible. Background noise levels were measured at 54–67 dB(C). Each animal was individually recorded over a duration of about 5–20 min, capturing the animals’ spontaneous vocalizations while being gently held by animal care staff. A Sennheiser MKE platinum-C microphone (Sennheiser Electronic GmbH & Co. KG, Wedemark, Germany) was positioned at a distance of 30 cm from each animal’s mouth.

In order to assess the nature of laryngeal voice production in the investigated animals, time-synchronized EGG signals [30] were acquired in parallel with the acoustic signals. EGG is a non-invasive method to monitor vocal fold vibration. A high-frequency, low-voltage current is passed between two electrodes on each side of the larynx at the level of the vocal folds. Intra-cycle variation of vocal fold contact—induced by vocal fold contacting and de-contacting at the frequencies of the produced sounds—causes time-varying electrical admittance across the two electrodes. The resulting EGG signal correlates well with the relative vocal fold contact area (VFCA) [31]. In this study, the EGG signals were captured with a Glottal Enterprises EG2-PCX (Syracuse, NY). Conductive gel was applied to the EGG electrodes as well as to the animals’ neck at the position of the thyroid cartilage. The EGG electrodes were held in place manually throughout the recording session. The quality of the EGG waveform was constantly monitored in real-time using a period-synchonous computer display.

The EGG and acoustic signals were simultaneously recorded and digitized using a Marantz PMD661MK II Handheld Solid-State Recorder (Cumberland, RI) operated at a sampling frequency of 48 kHz. The digitized signals were stored in 16-bit uncompressed WAV format.

#### (iii) *Ex vivo* data acquisition

The larynges of two male tufted capuchin monkeys (*Sap. apella*) were investigated in an excised larynx set-up [32]. The laryngeal samples were excised from fresh cadavers just after euthanasia of subjects with no hope of recovery (Ca12 and 18) as a veterinary procedure at the Primate Research Institute (PRI), Kyoto University, according to the 3rd edition of the Guide for the Care and Use of Laboratory Primates of the PRI (2010). One (specimen Ca12, labelled EL1 here) was a 29 year old male, and the other (specimen Ca18, labelled EL2 here) was a 25 year old male animal. The larynges of both animals were harvested immediately post-mortem. They were flash-frozen with liquid nitrogen and stored at −30°C. Immediately before the experiment the larynges were slowly thawed. The trachea of each larynx was shortened to about 2 cm to enable vertical mounting of the larynx on an air-supply tube.

Humidified and heated air was blown through each larynx from below, in order to induce self-sustained oscillation of the laryngeal tissue. Compressed air was produced by a Hitachi SC820 Silent Air Compressor (Hitachi Koki, Tokyo, Japan). The ensuing airflow was regulated by a Fairchild 10 202U Pneumatic Precision Regulator (Fairchild Industrial Products Company, Winston-Salem, NC, USA) and a CMQ-V digital mass flow controller (Azbil Corporation, Kyoto, Japan). The CMQ-V mass flow controller was also used to record air flow rates. Subglottal pressure was varied in a range of 1 kPa to 1.6 kPa, measured 32 cm upstream of the vocal folds with a PDS−70GA pressure probe (Kyowa Electronic Instruments Co. Ltd., Tokyo, Japan), amplified with a CDV−700A signal conditioner (Kyowa Electronic Instruments Co. Ltd., Tokyo, Japan). Sound pressure levels (not reported here) were monitored with a Brüel & Kjær 2250 sound level meter located 43 cm from the vibrating vocal folds, reporting A-weighted levels. The background noise levels were at 42 dB(A). The acoustic signal was recorded with a 4192 L-001 pressure field microphone (Brüel & Kjær, Nærum, Denmark), located at a distance of 22 cm from the vocal folds. The microphone signal was amplified with a 2669 signal preamplifier and a Nexus conditioning amplifier (both Brüel & Kjær, Nærum, Denmark). EGG data were acquired with an EGG-A100 laryngograph microprocessor (Laryngograph Ltd., London, UK). All signals were simultaneously acquired and digitized with a PXI−6143 multifunction I/O module connected to PXIe−8840 QuadCore embedded controller (both National Instruments, Austin, TX, USA) at a sampling rate of 50 kHz.

High-speed video (HSV) recordings of the vibrating vocal folds were recorded with a FASTCAM NOVA S16 32 GB camera (Photron, Tokyo, Japan). To ensure time-synchronized recording of the video data and all other signals, a transistor transistor logic (TTL) signal was used to trigger the video camera. As a redundant measure, this TTL signal also triggered a VW−760N wireless TTL flash (Gradus Group LLC, New York, NY) shortly after the beginning and before the end of each recording, and the respective light bursts were visible in the video footage. The TTL trigger signal was recorded by the NI data acquisition system, in synchrony with all other data.

Preliminary manual tests with the larynx specimens revealed that displacement of the arytenoids towards a posterior (dorsal) and slightly lateralized direction resulted in the medialization of the vocal membranes. In order to systematically test this phenomenon, a ‘hands-off’ investigative paradigm was established as follows: sutures were placed through the posterior parts of both the arytenoids individually, and non-elastic threads were connected from these sutures to a MLTF500/ST force transducer (ADInstruments, Nagoya, Japan). The force transducer was connected to a 28BYJ−48 stepper motor (Osepp, British Columbia, Canada), through which the tension within the threads was controlled. The direct mechanical connection from the servo motor—via the force transducer and the threads—to the arytenoids allowed for monitoring of the posterior elongation force of the arytenoids with the force transducer.

### (b) Data analysis

#### (i) *In vivo* data

Time-domain waveforms and narrow-band spectrograms of all captured acoustic and EGG signals were visually assessed. A total of 1375 vocalization samples produced by 12 individuals from six different species were found in the entire data corpus. After careful inspection, only those samples were retained where (i) animal vocalization was clearly identifiable in both the time-domain and the frequency-domain representation of the EGG signal; (ii) no other vocalization source (e.g. from animal care personnel or other animal’s sounds) was audible in the acoustic signal; and (iii) the vocalization systematically contained repeated and abrupt $f_{o}$ jumps between two distinct nearly periodic oscillatory regimes. In this manner, a total of 64 high-quality recorded samples with abrupt $f_{o}$ jumps were identified, stemming from five animals (2 x *Sap. apella*, 2 x *At. chamek*, 1 x *C. albifrons*—see the electronic supplementary material, table S3). These samples were further analysed as follows:

Within each sample, the low-frequency (low- $f_{o}$ ) and the high-frequency (high- $f_{o}$ ) portions were identified. Within both the low- $f_{o}$ and the high- $f_{o}$ portion, a sequence of consecutive glottal cycles was annotated manually with the Praat analysis framework [33], applying a peak-picking criterion. This particular analysis approach was chosen because the *in vivo* vocalizations typically exhibited rapid alterations between the low- $f_{o}$ and the high- $f_{o}$ states, often comprising only a few oscillatory cycles each. Notably, preliminary tests with the algorithmic autocorrelation analysis method used for *ex vivo* data (see below) did not produce reliable results, because the autocorrelation time-domain method requires periods of quasi-stable oscillation.

The 64 analysed samples and the respective cycle annotations are available as the electronic supplementary material. For each sample, the $f_{o}$ of the annotated low- $f_{o}$ and the high- $f_{o}$ portions were estimated by averaging the periods of the respective cycles and taking the reciprocal as


(2.1) $$f_{o}=\frac{n}{\sum T_{k}}\;\;\;[Hz],$$

where k is the cycle index, T is the cycle period and n is the number of cycles in the annotated sequence. To evaluate the relative magnitude of the systematic frequency transition within each sample, the dimensionless ratio of low- $f_{o}$ versus high- $f_{o}$ was simply computed as $r=f_{o:\text{high}}/f_{o:\text{low}}$ .

#### (ii) *Ex vivo* data

$f_{o}$ of the acoustic signal captured during the excised larynx experiments was computed with the autocorrelation method [33] included in the Praat analysis framework [34]. The raw high-speed video data were converted to the uncompressed AVI format using the Python mraw reader provided by Javh *et al* [35]. Digital kymograms [36] of selected portions of the AVI videos were then created with a custom script written in the Python programming language by C.T.H., which was used as a plugin within the FIJI image analysis framework [37]. For creating the kymograms, a line perpendicular to the glottal midline (positioned at the maximum vibratory amplitude of the vocal folds) was selected within the video footage. For each video frame, the colour intensities along the selected line were considered, and resulting kymographic lines (one for each video frame) were rotated vertically and then consecutively stacked horizontally to generate the kymogram image. In a kymogram, time is thus mapped onto the *x*-axis, and the time-varying medio-lateral deflections of the laryngeal tisse are shown on the *y*-axis.

### (c) Computational ‘Neubauer’ model

To reproduce the excised larynx experiment, a mathematical model of the vocal folds and the vocal membrane was used, originally based on previous work by Mergell *et al*. [38]. In that model, the vocal folds are represented by a ‘classic’ two-mass system, composed of lower and upper masses ( $m_{1}$ , $m_{2}$ )—see the electronic supplementary material, figure S7 for a schematic illustration. On the upper mass, a vocal membrane is attached as a static reed-like plate which vibrates with the vocal fold. This model was further developed by Neubauer [27], implementing the possibility for a time-varying angle of the vocal membrane, thus adding an extra degree of freedom by supporting the independent oscillation of the vocal membrane with respect to the other two masses. This additional degree of freedom is needed to simulate different laryngeal mechanisms with respect to the involvement of the vocal membranes (a notion that was not investigated by Mergell *et al*., who mainly concentrated on phonation threshold pressure and glottal efficiency [38]). Here, we implement Neubauer’s version of the model.

The ‘Neubauer’ model was created with the following assumptions:

(i) the vocal folds and vocal membranes show symmetric movements between the left and right sides;
(ii) effects of the sub- and supra-glottal resonances can be ignored;
(iii) below the narrowest part of the glottis, the intra-glottal pressure obeys Bernoulli’s principle; and
(iv) collision forces, which may arise during the contact of the left and right vocal membranes, are neglected.

The model’s equations are given by:


(2.2) $$m_{1}{\ddot{x}}_{1}+r_{1}{\dot{x}}_{1}+k_{1}x_{1}+k_{c}(x_{1}-x_{2})-\Theta (-a_{1})c_{1}\frac{|a_{2}|}{2L}=Ld_{1}P_{1},$$

$$\left(m_{2}+m_{3}){\ddot{x}}_{2}+r_{2}{\dot{x}}_{2}+k_{2}x_{2}+k_{c}(x_{2}-x_{1})-\frac{1}{2}m_{3}d_{3}(\ddot{\theta }\cos\theta_{a}-{\dot{\theta }}^{2}\sin\theta_{a}\right)$$

(2.3) $$-\Theta (-a_{2})c_{2}\frac{|a_{2}|}{2L}=Ld_{2}P_{2}+L\int_{0}^{d_{3}\cos\theta_{a}}P_{3}(y)dy,$$

(2.4) $$\frac{1}{3}m_{3}d_{3}^{2}\ddot{\theta }+r_{3}\dot{\theta }+k_{3}(\theta +\eta \theta^{3})-\frac{1}{2}m_{3}d_{3}{\ddot{x}}_{2}\cos\theta_{a}=-\frac{L}{\cos^{2}\theta_{a}}\int_{0}^{d_{3}\cos\theta_{a}}yP_{3}(y)dy.$$

The dynamical variables $x_{i}(t)$ represent displacements of the two masses ( i=1 : lower mass, i=2 : upper mass), while $\theta_{a}(t)=\theta_{0}+\theta (t)$ is the angle of the vocal membrane with respect to the upper mass ( $\theta_{0}$ : pre-phonatory angle, θ : deviation from $\theta_{0}$ ). The parameters $m_{i}$ , $r_{i}$ , $k_{i}$ , $c_{i}$ and $d_{i}$ represent weight, damping, stiffness, collision stiffness and thickness of the two masses ( i=1,2 ) and the vocal membrane ( i=3 ), respectively, while the parameter $k_{c}$ represents a coupling between the two masses. The collision function is approximated as Θ(x)=0 ( x≤0 ); Θ(x)=tanh⁡(1000x) ( 0<x ). The glottal areas of the lower and upper masses are given by $a_{i}=2L\{x_{0i}+x_{i}\}$ ( i=1,2 ), where $x_{0i}$ represents the prephonatory distance and L is the glottal length. The area function along the vocal membrane is given by $a_{3}(y)=a_{2}-2L\;y\;\tan\theta_{a}$ ( $0\leq y\leq d_{3}\cos\theta_{a}$ ), while the area at the tip of the vocal membrane is $a_{vm}=a_{3}(d_{3}\cos\theta_{a})=a_{2}-2Ld_{3}\sin\theta_{a}$ . The opening area of the vocal folds is determined as $a_{vf}=\min(a_{1},a_{2})$ , and the narrowest opening area of the glottis is given by $a_{min}=\min(a_{1},a_{2},a_{vm})$ . The pressures $P_{1}$ , $P_{2}$ , $P_{3}$ —which act on the right–hand–side of equations (2.2)–(2.4)—as well as the glottal volume flow are obtained from Bernoulli’s principle as:


(2.5) $$P_{1}=P_{s}[1-(\frac{a_{\text{min}}\Theta (a_{\text{min}})}{a_{1}}{)}^{2}]\Theta (a_{1}),$$

(2.6) $$P_{2}=P_{s}[1-(\frac{a_{\text{min}}\Theta (a_{\text{min}})}{a_{2}}{)}^{2}]\Theta (a_{1})\Theta (a_{2})\Theta (a_{1}-a_{vm})\Theta (a_{2}-a_{vm}),$$

(2.7) $$P_{3}(y)=P_{s}[1-(\frac{a_{\text{min}}\Theta (a_{\text{min}})}{a_{3}(y)}{)}^{2}]\Theta (a_{1})\Theta (a_{2})\Theta (a_{1}-a_{vm})\Theta (a_{2}-a_{vm}),$$

(2.8) $$U=\sqrt{\frac{2P_{s}}{\rho }}a_{\text{min}}\Theta (a_{\text{min}}),$$

where $P_{s}$ represents the subglottal pressure and ρ is the air density.

To simulate the dynamics of the vocal folds and vocal membranes, the parameter values were adopted from the standard ones used in [27,39] as: $m_{1}=0.0833$ g, $m_{2}=0.0167$ g, $m_{3}=0.0017$ g, $d_{1}=0.25$ cm, $d_{2}=0.05$ cm, $d_{3}=0.05$ cm, $k_{1}=0.12$ g ms^−2^, $k_{2}=0.012$ g ms^−2^, $k_{c}=0.0375$ g ms^−2^, $k_{3}=9.2\cdot 10^{-6}$ g cm ${,}^{2}$ ms ${\;}^{-2}$ , η=1 , $c_{1}=3k_{1}$ , $c_{2}=3k_{2}$ , $r_{1}=0.02$ g ms^−1^, $r_{2}=0.02$ g ms^−1^, $r_{3}=7.23\cdot 10^{-8}$ g cm ${,}^{2}$ ms^−1^, $\theta_{0}=0.01\pi$ rad, L=1.4 cm, ρ=0.0013 g cm ${\;}^{-3}$ and $P_{s}=0.8$ kPa. The differential equations were solved by using the *ode23* solver in MATLAB (R2021b; MathWorks, Natick, MA, USA).

To simulate the register-like transitions (or ‘yodels’), switching between two oscillation modes is important: (i) oscillations of only the vocal membrane versus (ii) oscillations of both vocal folds and vocal membranes. These oscillatory modes can be regulated by adjusting the prephonatory distance of the lower parts of the vocal folds $x_{01}$ . For a small $x_{01}$ , the entire vocal folds are abducted and the vocal folds and the vocal membranes vibrate together. For a large $x_{01}$ , the lower parts of the vocal folds are abducted and do not participate in the vocal fold oscillations, where only the vocal membranes (and upper parts of the vocal folds) vibrate. In the rotational coordinate, the prephonatory distances of the lower and upper parts of the vocal folds are given by $x_{01}=r\{1-\cos(\alpha_{1}+\beta )\}$ and $x_{02}=r\{1-\cos(\alpha_{2}+\beta )\}$ , where $\alpha_{1}=-(30/180)\pi$ rad, $\alpha_{2}=0$ rad, and r=1 cm.

For the simulation, the Neubauer model was oscillated for 4 s, during which the angle β was gradually changed from (15/180)π rad to (5/180)π rad. Then it was oscillated for another 4 s, during which the angle β was gradually changed back to (15/180)π rad.

## 3. Results

### (a) Anatomy

**Figure 2.**
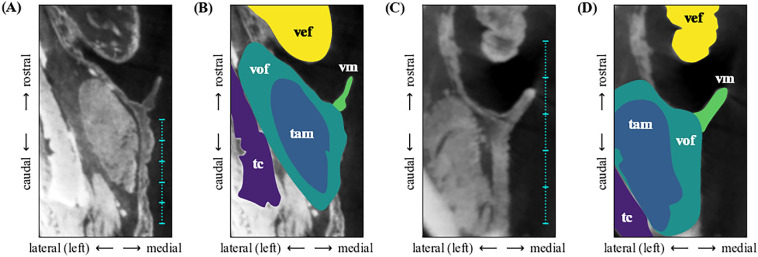
Micro-CT scans of two New World monkey larynges: *Ateles paniscus* (A and B) and *Sapajus apella* (C and D), left side of larynx shown. Selected anatomical structures are highlighted in panels (B) and (D). The thyroarytenoid muscle (tam) makes up the major bulk of the vocal fold (vof). The vocal membranes (vm) are depicted in light green colour. The vertical scales in panels (A )and (C) each represent a spatial extension of 5 x 1 mm. vef: ventricular fold; tc: thyroid cartilage.

The micro-CT scans depicted in figure 2 show the left half of the larynx of two species in sagittal section: *Ateles paniscus* and *Sapajus apella*. The vocal membranes appear as thin upward extensions of the superficial vocal fold layer [40] into the medio-rostral direction. This anatomical configuration is comparable to previously presented data [26] in non-human primates.

### (b) *In vivo* vocalizations

**Figure 3.**
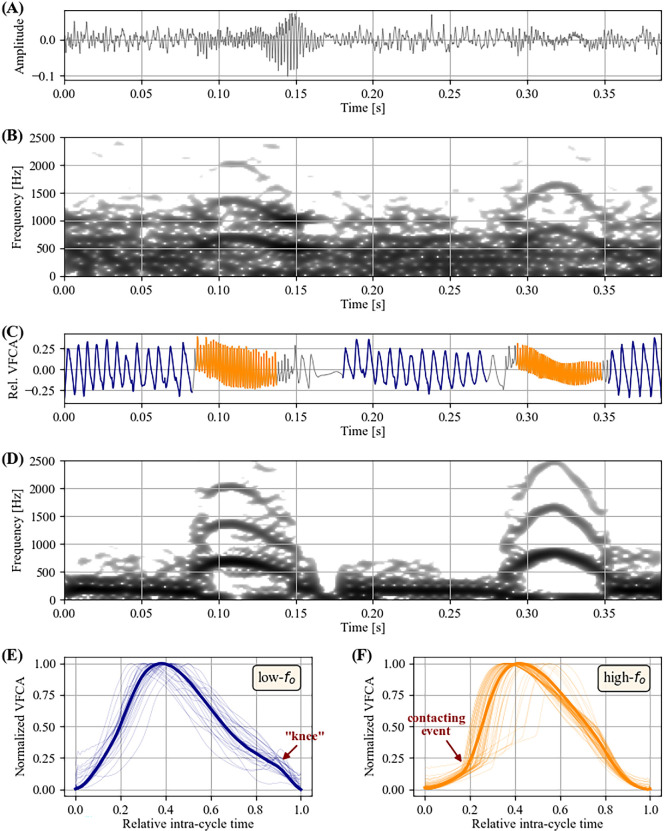
Prototypical ‘yodel’ vocalization of a Peruvian spider monkey (*Ateles chamek*), documented by acoustic (A) and electroglottographic (EGG) signals (C). (B) and (D) show narrow-band spectrograms of the acoustic and EGG signals, respectively (dynamic range = 38 dB). Two distinct laryngeal vibratory states—highlighted in dark blue (low- $f_{o}$ vibratory state, (C) and (E) and orange (high- $f_{o}$ vibratory state, (C) and (F) colours—are clearly discernable in the EGG signal. See text concerning the annotations in (E) and (F).

A representative example of a ‘yodel’ phonation with repetitive abrupt $f_{o}$ jumps, produced by a Peruvian spider monkey (*At. chamek*), is documented in figure 3. The EGG signal reveals a systematic alternation of two vibratory mechanisms, one for low- $f_{o}$ (annotated in dark blue colour) and the other for high- $f_{o}$ (orange)—see figure 3C,E and F. The two respective EGG waveforms (panels E and F) show systematic differences. The the low- $f_{o}$ mechanism produces a so-called ‘knee’ (i.e. a convex discontinuity during the descending part of the waveform [41]) late in the cycle, while no such pronounced feature is seen in the high- $f_{o}$ mechanism. By contrast, the high- $f_{o}$ mechanism is characterized by a sharp discontinuity at around 20% of the relative cycle duration, probably representing the moment when the vocal fold contact is abruptly increasing [31], while the low- $f_{o}$ mechanism shows a more gradual increase of vocal fold contact without such a discontinuity.

The transition between these two mechanisms occurs within a few glottal cycles and is thus indicative of a bifurcation phenomenon in the context of nonlinear dynamics. In contrast to the EGG signal, these bifurcations between two distinct laryngeal mechanisms are not well discernible in the acoustic signal (panels A and B), which is probably owing to the influence of background noise. In particular, the acoustic information of the low- $f_{o}$ mechanism — audible to human ears when close — was obscured by acoustic artefacts. If similar phenomena also occur in other field recordings that document acoustic signals alone, this might explain why abrupt $f_{o}$ jumps—and thus the notion of vocal registers in non-human mammals in general—have to date not received much scientific attention.

**Figure 4.**
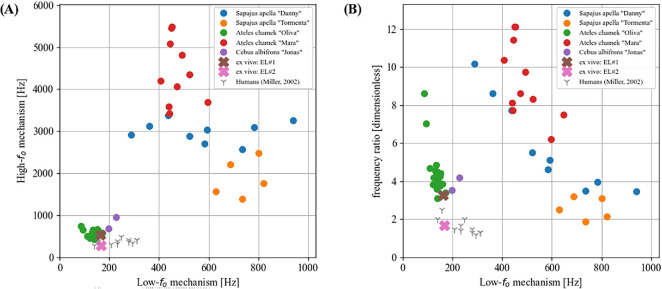
Synoptic $f_{o}$ bifurcation data of all analysed vocalizations, computed from the respective EGG signals. (A) respective frequencies of low- $f_{o}$ vibratory mechanism in relation to the high- $f_{o}$ vibratory mechanism for each analysed vocalization. (B) same as (A), but showing the frequency ratio between the low- $f_{o}$ versus the high- $f_{o}$ vibratory mechanisms on the ordinate. Analysis data of *in vivo* recordings are plotted as filled circles. Data from the two excised tufted capuchin monkey larynges (*Sapajus apella*) are shown with crosses. Data of characteristic leap intervals between M1 and M2 vocal registers in human singers [16] are indicated with small grey Y markers.

A synoptic overview of $f_{o}$ jumps observed in the collected *in vivo* data is shown in figure 4 (see also the electronic supplementary material, figure S8 for a comparison with previously documented $f_{o}$ jumps in New World monkeys). In figure 4A, the fundamental frequency of the low- $f_{o}$ mechanism is plotted against the respective high- $f_{o}$ mechanism for all 64 analysed vocalization samples. The $f_{o}$ data gained from the excised larynx (*ex vivo*) experiment are displayed in the same fashion. ‘Characteristic leap intervals’ of human singers [16], alternating between the M1 and M2 vocal registers, are indicated for the purpose of comparison. Panel B shows the same data, but displaying the relative frequency ratio between the low- $f_{o}$ and the high- $f_{o}$ mechanism for each call on the ordinate. Human register transitions are largely limited to a frequency ratio of 2 or below, i.e. with one exception never exceeding a doubling of $f_{o}$ (an increase of an octave in terms of musical intervals) when changing from M1 to M2. By contrast, most $f_{o}$ jumps observed *in vivo* in monkeys are beyond a frequency ratio of 2, in one extreme case resulting in a factor of 12 (comparable to a musical interval of 43 semi-tones or about three and a half octaves). The data obtained from the excised larynx experiment for larynges EL1 and EL2 are comparable with the $f_{o}$ ratios found for the spider monkey and white-fronted chapuchin monkey *in vivo*, but lower in frequency than the two tufted chapuchin monkeys. This may be attributed to the fact that in the excised larynx experiment only a limited amount of vocal fold elongation—simulating the action of the cricothyroid muscle—could be performed during data acquisition by posteriorly displacing the arytenoids (see Methods), while cricothyroid muscle activation *in vivo* might result in stronger effects during vocalization.

EGG signal analysis showed systematic amplitude differences for low- $f_{o}$ versus high- $f_{o}$ mechanisms see the electronic supplementary material, figure S9. Such overall amplitude differences have been found to be typical for transitions between the M1 and M2 vocal registers in humans [19]. Given that the EGG signal is a correlate of the relative vocal fold contact area [31], this indicates that the high- $f_{o}$ mechanism was produced with a reduced vocal fold contact area. This suggests that different laryngeal structures may have been contributing to the low- $f_{o}$ versus the high- $f_{o}$ mechanism.

### (c) Excised larynx experiment

**Figure 5.**
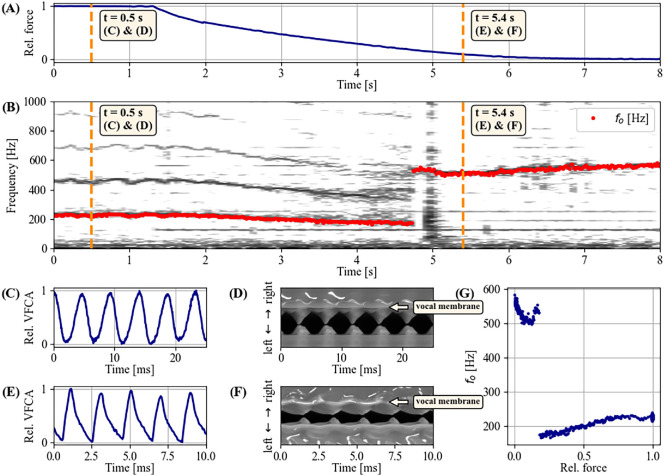
*Ex vivo* (excised larynx) data of a tufted capuchin monkey larynx (specimen EL1): overall $f_{o}$ control is facilitated by vocal membrane adduction via force-elongated arytenoid rotation. (A) Relative posterior (dorsal) arytenoid displacement force (servo-motor controlled); (B) narrow-band spectrogram of acoustic signal, with superimposed $f_{o}$ data as red dots. At around *t* ≈ 4.8 s the oscillation abruptly changes from the low-frequency mode (without vocal membranes) to the high-frequency mode (with entrained vocal membranes—see text). Starting at *t* ≈ 1.3 s the artefactual noise of the utilized servo motor is visible in the spectrogram, with a constant $f_{o}$ of 124 Hz and subharmonics every 62 Hz. (C) and (D) EGG signal and respective kymogram from HSV footage for the low- $f_{o}$ vibratory state, extracted at *t* = 0.5 s. Note that the vocal membranes are not participating in the laryngeal oscillation; (E) and (F) EGG and kymographic data for the high- $f_{o}$ vibratory state extracted at *t* = 5.4 s, showing vibrating vocal membranes in (F); (G) bifurcation diagram for $f_{o}$ control via arytenoid rotation.

Data from the excised larynx preparation are presented in figure 5. In contrast to most other excised larynx investigations [32], artificial adduction of the arytenoids was not required for initiation of self-sustaining oscillations of the laryngeal tissue. Posterior (dorsal) and slightly lateral displacement of the arytenoids (see Methods) led to a rotational rocking motion of the arytenoids, resulting in lateralization of the vocal membranes. With the vocal membranes lateralized (*t* = 0 to 4.8 s), they did not vibrate, or obviously participate in laryngeal oscillation (see panel D for a digital kymogram). The restoration of the arytenoids to their original position by relaxing the posterior elongation force (see panel A) resulted in an abrupt shift of the oscillatory mechanism visible at *t* ≈ 4.8 s in figure 5B, coinciding with an abrupt upward shift of $f_{o}$ from about 163 Hz to about 534 Hz— i.e. a factor of 3.27 or about 1.71 musical octaves. In contrast to the low- $f_{o}$ mechanism (where vocal membranes did not oscillate; panel D), the vocal membranes oscillated in synchrony with the vocal folds in the high- $f_{o}$ mechanism (panel F). The $f_{o}$ data for the two observed oscillatory states are indicated as a brown X in figure 4A,B, labelled EL1. Data from a trial with a second larynx, EL2 (illustrated in the electronic supplementary material, figure S10), are depicted with a pink X in figure 4A,B. Notably, EL2 exhibited an $f_{o}$ increase of only 70 % when switching from the low- $f_{o}$ to the high- $f_{o}$ state. We thus cannot rule out that the bifurcation observed in EL2 was—in contrast to the bifurcation found in EL1—mainly driven by individual eigenmodes of vocal folds, just as observed in the ‘classical’ register transitions in humans (see [42] for a deeper discussion).

### (d) Computational ‘Neubauer’ model

**Figure 6.**
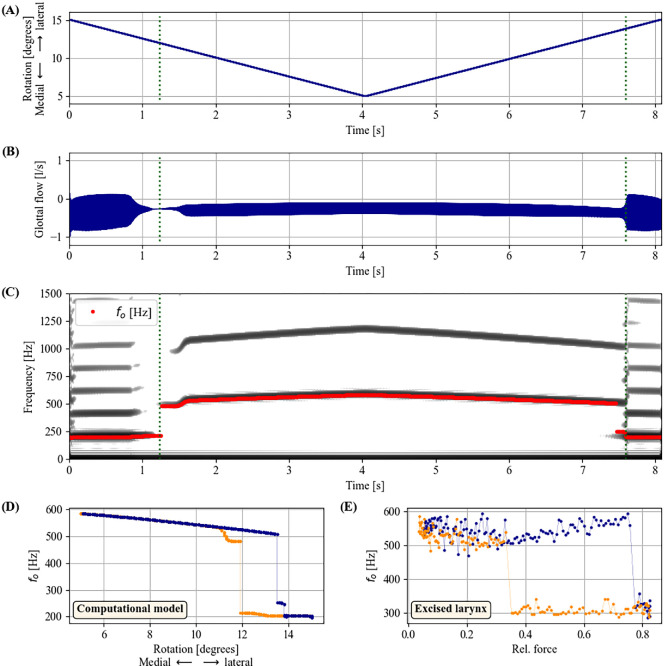
$f_{o}$ bifurcations and hysteresis as produced by the computational model. (A) vocal membrane adduction parameter (see Methods and text); (B) time-varying glottal air flow; (C) narrow-band spectrogram of glottal air flow. The bifurcations betwen the low- $f_{o}$ and high- $f_{o}$ mechanisms in panels (A)–(C) ( t≈1.2 s and t≈7.6 s) are indicated with dark green dotted vertical lines; (D) hysteresis in computational model: bifurcation diagram of vocal membrane adduction vs. oscillatory frequency ( $f_{o}$ ). Data for increasing and decreasing adduction are plotted in blue and orange, respectively; (E) hysteresis in excised larynx data: bifurcation diagram from *ex vivo* data of larynx specimen EL1.

The results from the excised larynx investigation were corroborated with a lumped-mass oscillatory model (figure 6). In the model, the adduction of the vocal membrane was controlled by a slow angular rotation of the vocal membrane and the upper mass (see Methods), thus mimicking the rotational lateral displacement motion observed in the arytenoid when pulling them posteriorly. In particular, a low degree of rotation positioned the upper mass (M1) of the left and right vocal folds more medially (i.e. further apart from each other), preventing the lower mass (M1) from participating in the laryngeal oscillations. In this condition, only the upper mass (M2) and vocal membranes vibrated. By contrast, a higher degree of rotation adducted the vocal folds, so that they vibrated as the main oscillator. There, vocal membranes were entrained to the vocal fold oscillations.

As can be seen in figure 6A–C, the oscillation clearly jumped between a low- $f_{o}$ and a high- $f_{o}$ mechanism, analogous to what was observed *ex vivo* (recall figure 5). This corroborates the finding that the high- $f_{o}$ mechanism is facilitated by participation and entrainment of the vocal membrane during laryngeal sound generation, as suggested theoretically by Neubauer [27]. As is typical for vocal register transitions [3,16], the shift between the low- $f_{o}$ and a high- $f_{o}$ mechanisms exhibited a hysteresis phenomenon analogous to what is shown in figure 1B: when rotating the vocal membrane laterally, the bifurcation and $f_{o}$ jump occurred at a greater roational angle of $\beta \approx 14{,}^{\circ }$ as compared to the medial rotation of the vocal membrane, where the bifurcation occurred at $\beta \approx 12{,}^{\circ }$ (figure 6D). This is again comparable to what was observed in the excised larynx set-up, where the hysteresis effect was brought about by bifurcation between the low- $f_{o}$ and high- $f_{o}$ mechanisms at different posterior arytenoid extensions (figure 6E), depending on the arytenoid rotation direction (lateral versus medial). In terms of nonlinear dynamics, this hysteresis phenomenon is created by a cusp catastrophe formed by two saddle-node bifurcations of limit cycle oscillators, one for increasing and one for decreasing $f_{o}$ . Notably, such saddle-node bifurcations (and the resulting hysteresis) are also considered to be the main cause for register transitions and $f_{o}$ jumps in human voice production [3,43].

## 4. Discussion

The key finding of this study is that voice production in New World monkeys is facilitated by two distinct laryngeal mechanisms, analogous to human vocal registers: a low- $f_{o}$ mechanism produced by vocal fold oscillation; and a high- $f_{o}$ mechanism, which is causally enabled by oscillation and entrainment of the vocal membranes, thin anatomical upward extensions of the vocal folds [26]. *Ex vivo* and computational modelling data suggest that these two laryngeal mechanisms may be controlled by arytenoid rotation, resulting in medialization (for the high- $f_{o}$ mechanism) or lateralization (for the low- $f_{o}$ mechanism) of the vocal membranes. Gradual adjustment of arytenoid position (and thus the vocal membrane position along the medio-lateral dimension) results in bifurcations between the two laryngeal mechanisms, i.e. abrupt changes and hysteresis phenomena between oscillatory states at distinctly different $f_{o}$ , again analogous to human vocal registers. The excised larynx findings constitute evidence against the theoretical possibility that the abrupt transitions between the observed laryngeal mechanisms were brought about by rapid controlled parameter changes via super-fast laryngeal muscles, as has been found in songbirds [44].

In humans and one canine *ex vivo* model, activation states of intrinsic laryngeal muscles (predominantly the thyroarytenoid muscle embedded in the vocal fold) were shown to be major influence factors for transitions between the M1 and M2 laryngeal mechanisms [45–47]. Given comparable laryngeal anatomy, it is likely that the laryngeal setup of non-human primates possesses a similar control structure. However, the frequency ratios between the low- $f_{o}$ and the high- $f_{o}$ mechanism in our data far exceed what is observed in humans (recall figure 4B), where characteristic leap intervals between the M1 and the M2 vocal registers typically are below one octave. We propose that the extreme $f_{o}$ jump ratios we have documented in New World monkey ‘yodels’ are crucially facilitated by the involvement of their pronounced, thin vocal membranes, an anatomical feature typical of primates that has been lost in human evolution (perhaps to allow for the production of more stable, harmonic-rich, phonation for speech) [26]. A similar argument might be made for bats who also possess a vocal membrane [38].

In our experimental set-up, we introduced the medio-lateral rotation of the arytenoids—and thus the positioning of the vocal membranes—via oblique posterior displacement of the arytenoids. This posterior displacement had an additional, elongating effect on the vocal folds, slightly increasing $f_{o}$ as a function of elongation force. Note that the decrease of $f_{o}$ in synchrony with the decrease of elongation force continued even after the abrupt upward jump of $f_{o}$ in figure 5B, suggesting two superimposed agents of $f_{o}$ control: (i) vocal fold elongation (gradual); and (ii) bifurcation between oscillatory mechanisms (abrupt). In analogy to human voice production [48,49], (iii) subglottal pressure (not reported here) presumably constitutes a third means of control. Partly contrasting our findings, Zhang *et al*. found that the drastic changes of $f_{o}$ in the marmoset larynx were solely brought about by variation of vocal fold strain, i.e. changes of vocal fold elongation [50]. This might suggest that different laryngeal anatomy with respect to vocal membrane position and shape may result in a different control mechanism in non-human primates.

Given the available data, we are unable to say whether transitions between the low- $f_{o}$ and the high- $f_{o}$ mechanism necessarily always result in bifurcations, or whether the vocalizing animals may also possess the ability to smoothly transition between the two mechanisms, as in ‘voix mixte’ in human singing [51,52], or transitions between the low-frequency and high-frequency sides of the syrinx in some songbirds [53]. Given the larger observed frequency differences in our data *in vivo*, it may be hypothesized that such smooth transitions may be harder to maintain when vocal membranes are involved.

As regards the frequency ranges of $f_{o}$ jumps, both the *in vivo* and *ex vivo* data presented here are comparable with data found distributed in a number of previous studies—cf. electronic supplementary material, figure S8 and table S1. Notably, however, the systematic nature of the $f_{o}$ jumps and their different laryngeal production mechanisms has not been considered in most previous studies. In comparison to humans, $f_{o}$ jumps and bifurcations in the vocalizations of non-human primates have received relatively little previous scientific attention, some important landmark publications notwithstanding (e.g [4], as well as table 1 and §5.1 in [10]). This might have been owing to methodological issues during field experiments: typically only acoustic evidence is acquired, and background noise may often mask the low- $f_{o}$ components of calls, thus obscuring the $f_{o}$ transitions. This phenomenon was observed in our *in vivo* data, where the true nature of the $f_{o}$ bifurcations was manifest only in the EEG evidence (recall figure 3), capturing laryngeal dynamics at the source.

In addition to providing a theoretical and empirical framework for understanding distinct laryngeal mechanisms involving the vocal membranes, our findings clearly corroborate the previously suggested notion of vocal registers in New World monkeys—cf. Fig. 5C in [23]. These $f_{o}$ jumps greatly surpass by far what is possible with the human voice, highlighting a remarkable and as yet uninvestigated aspect of non-human primate vocal communication. Importantly, we hypothesize that different call types in non-human primate species may—among others factors—be supported by distinct laryngeal mechanisms. This is in line with data from Herbst *et al*. who recently showed with EGG evidence that the coo, growl, and chirp calls of a Japanese macaque were produced using different laryngeal mechanisms, analogous to vocal registers in humans [54]. The evidence presented in these studies indicates, for the first time to our knowledge, how variation in larynx anatomy may serve as a means of increasing complexity in the emitted vocalizations using the peripheral anatomy rather than central nervous control—cf. [55]. This capacity may have evolved specifically to enrich the animal call repertoire, potentially used for attention grabbing changes, avoidance of habituation, call diversification and/or signaling of individuality.

## 5. Conclusion

We show that abrupt $f_{o}$ jumps and bifurcations are systematically present in the vocalizations of New World monkeys, and that they are causally driven by distinct laryngeal vibratory mechanisms, analogous to vocal registers in humans. The low- $f_{o}$ versus high- $f_{o}$ mechanism are facilitated by involvement (or lack thereof) of the vocal membranes in vocal fold oscillation, potentially controlled by a single smoothly varying parameter of arytenoid rotation. We conclude that—in non-human primates and probably other animals—different call types may be powered by distinct laryngeal mechanisms analogous to vocal registers in humans.

These findings and conceptual insights may pave the way for further research regarding the communicative purpose of abrupt and large $f_{o}$ transitions. We found that $f_{o}$ jumps in nonhuman primates far exceed (up to five times larger) the frequency leaps seen in human register transitions, suggesting that non-human primates use peripheral anatomical mechanisms to increase the complexity of their vocal repertoire. For human speech, where abrupt register transitions are typically avoided, a more simple laryngeal ‘carrier signal’ is needed. It appears that the evolutionary loss of vocal membranes has allowed human speech to avoid non-linear phenomena such as abrupt register transitions [26]. By contrast, perhaps singing—where vocal registers are amply used, sometimes even with deliberate bifurcations between them (e.g. in yodelling)—still enables us to exploit the acoustic richness of our primate heritage .

## Data Availability

All Materials can be downloaded in one large ZIP file (ca 100 MB) from: http://www.christian-herbst.org/monkey_yodels/supplMat.zip. Supplementary material is available online [56].
